# Diagnostic performance of gated SPECT myocardial perfusion imaging for early detection of diastolic dysfunction in diabetic patients: A comparative study with echocardiography

**DOI:** 10.22038/aojnmb.2026.92104.1676

**Published:** 2026

**Authors:** Reyhane Ahmadi, Soheib Mardokhi

**Affiliations:** Farshchian Heart Center, Hamedan, Iran

**Keywords:** Diabetic Cardiomyopathy Gated SPECT, Myocardial Perfusion Imaging Echocardiography, Peak Filling Rate (PFR)

## Abstract

**Objective(s):**

Diabetic patients are at high risk for developing diabetic cardiomyopathy, often initially presenting as left ventricular diastolic dysfunction (LVDD). While echocardiography is the standard for diagnosing LVDD, Gated SPECT (GSPECT) offers simultaneous assessment of perfusion and function. This study aimed to evaluate the diagnostic value of diastolic parameters derived from GSPECT for detecting LVDD in diabetic patients without known heart disease, using echocardiography as a reference.

**Methods:**

In this cross-sectional study, 60 diabetic patients without a history of cardiac disease underwent both GSPECT myocardial perfusion imaging and echocardiography within a two-week period. Diastolic function was assessed on GSPECT using peak filling rate (PFR) and time to peak filling rate (TTPFR). Echocardiography served as the gold standard for classifying diastolic function as normal, grade 1, or grade 2 dysfunction. Diagnostic accuracy, sensitivity, and specificity of GSPECT parameters were calculated.

**Results:**

Based on echocardiography, 51.7% of patients had LVDD (45% grade 1, 6.7% grade 2). The PFR derived from GSPECT demonstrated a sensitivity of 87% and a specificity of 48.2% for detecting LVDD, with an overall accuracy of 68.3%. TTPFR showed high sensitivity (90.3%) but low specificity (17.2%). A significant correlation was found between the ischemia pattern on GSPECT and the presence of diastolic dysfunction (p=0.01). Lower PFR values were significantly associated with male gender, age >60 years, and smoking.

**Conclusion:**

Gated SPECT, particularly the PFR parameter, shows high sensitivity for the detection of LVDD in asymptomatic diabetic patients. Incorporating diastolic function analysis into routine GSPECT reporting can provide valuable incremental information for the early diagnosis and risk stratification of diabetic cardiomyopathy.

## Introduction

 Cardiovascular disease is the leading cause of mortality in patients with diabetes mellitus (1). Beyond accelerating atherosclerosis, diabetes can directly damage the myocardium, leading to a specific entity known as diabetic cardio-myopathy (2). The earliest manifestation of this condition is often left ventricular diastolic dysfunction (LVDD), which can progress to overt heart failure with preserved ejection fraction (HFpEF) while patients remain asymptomatic (3,4).

 Early detection of LVDD is crucial for initiating timely interventions that may slow or reverse disease progression (5). Echocardiography, particularly with Doppler assessment, is the clinical gold standard for evaluating diastolic function (6). However, a significant proportion of diabetic patients undergo myocardial perfusion imaging (MPI) with Gated SPECT (GSPECT) for the assessment of coronary artery disease. GSPECT is primarily used to evaluate perfusion defects and left ventricular ejection fraction (LVEF), but it also provides quantitative parameters of diastolic function, such as the peak filling rate (PFR) and time to peak filling rate (TTPFR) (7,8). 

 While these diastolic indices are computationally available, they are often underutilized in clinical reporting. Previous studies have yielded inconsistent results regarding the correlation between GSPECT-derived diastolic parameters and echocardio-graphic findings (9, 10). Therefore, this study seeks to evaluate the diagnostic accuracy of GSPECT-derived diastolic indices for detecting LVDD in a cohort of diabetic patients without known cardiac disease, using comprehensive echocardiography as the reference standard.

## Methods

### Study Design and Population

 This cross-sectional study was conducted at the Farshchian Heart Center, Hamedan. Sixty diabetic patients without a prior history of heart disease, referred for clinically indicated GSPECT between 2021 and 2022, were enrolled via convenience sampling. The study was approved by the institutional ethics committee, and informed consent was obtained from all participants.

 Inclusion criteria were: 1) documented history of diabetes mellitus; 2) referral for MPI; and 3) willingness to undergo research echocardiography within two weeks of the GSPECT scan. Exclusion criteria included: 1) known history of coronary artery disease, heart failure, or valvular heart disease; 2) incomplete medical records; and 3) any change in clinical status between the two tests.

### Gated SPECT Imaging and Analysis

 Myocardial perfusion imaging was performed using a Siemens gamma camera with a two-day stress/rest protocol. Stress was induced either by treadmill exercise or pharmacological agents as clinically indicated. Gated SPECT images were acquired post-stress with 16 frames per cardiac cycle.

 Perfusion images were interpreted qualitatively by an experienced nuclear medicine physician for the presence and severity of ischemia (normal, 

mild, severity of ischemia (normal, mild, moderate, severe). Functional analysis was performed using automated software to calculate left ventricular ejection fraction (LVEF), end-diastolic volume (EDV), end-systolic volume (ESV), and diastolic indices. The peak filling rate (PFR) was expressed in end-diastolic volumes per second (EDV/s), and the time to peak filling rate (TTPFR) in milliseconds (ms). Based on prior literature, a PFR <2.5 EDV/s and a TTPFR >180 ms were considered indicative of diastolic dysfunction (9).

### Echocardiography

 All patients underwent comprehensive transthoracic echocardiography (Vivid E95, GE Healthcare) within two weeks of the GSPECT study. Studies were performed and interpreted by experienced cardiologists blinded to the GSPECT results. Diastolic function was assessed according to current ASE/EACVI guidelines (6), integrating mitral inflow Doppler, tissue Doppler imaging (e'), and left atrial volume index. Patients were subsequently classified as having normal diastolic function, grade 1 (impaired relaxation), or grade 2 (pseudo-normalized) dysfunction.

### Statistical Analysis

 Data were analyzed using SPSS software (Version 26.0). Continuous variables were expressed as mean±standard deviation (SD) and compared using Student's t-test or ANOVA. Categorical variables were presented as frequencies (percentages) and compared using the Chi-square test. The diagnostic performance (sensitivity, specificity, positive predictive value, negative predictive value, and overall accuracy) of GSPECT parameters (PFR, TTPFR, and LVEF) for detecting LVDD was calculated against the echocardiographic diagnosis. A p-value of < 0.05 was considered statistically significant.

## Results

### Patient Characteristics

 The study included 60 patients (51.7% female, mean age 63.96±12.50 years). The mean body mass index (BMI) was 26.4±2.3 kg/m², and the mean duration of diabetes was 9.3±5.25 years. Common cardiovascular risk factors were prevalent: 75% had hypertension, 51.7% were smokers, and 25% had uncontrolled diabetes (elevated HbA1c). GSPECT perfusion findings were normal in 43.3% of patients, while 56.7% showed evidence of ischemia (25% mild, 23.3% moderate, 8.3% severe) (Tables 1, 2).

**Table 1:** Distribution of variables of age, BMI, duration, and scan of diabetes

**Variable**	**N**	**Min**	**Max**		**Mean**	**SD**
Age	60	29	85		63.96	12.50
BMI	60	22	23		26.4	2.3
Duration of diabetes	60	1	20		9.3	5.25
**Scan variable**	**N**	**Min**	**Max**	**Mean**		**SD**
PFR^a^	60	1.0	5.12	2.07		0.78
TTPFR^b^	60	160	314	242.32		31.47
EF	60	40%	65%	55%		7.05

^a^PFR values <2.5 were considered positive

^b^TTPFR values >180 were considered positive

**Table 2:** Frequency of gender, diabetes control, history of hypertension, smoking, and heart perfusion status

**Variable**	**Category**	**F (%)**
Gender	Man	29(48.3%)
	Female	31 (51.7%)
History of HTN	Yes	45 (75%)
	No	15 (25%)
Diabetes control (HbA1c)	Controlled	45 (75%)
	Not controlled	15 (25%)
Smoking	Yes	31 ((51.7%)
	No	29 (48.3%0)
Heart perfusion status (based on scan)	Normal	26 (43.3%)
	Mild ischemia	15 (25%)
	Moderate ischemia	14 (23.3%)
	Severe ischemia	5 (8.3%)

### Echocardiographic and GSPECT Functional Findings

 Based on echocardiography, 29 patients (48.3%) had normal diastolic function, 27 (45%) had grade 1 dysfunction, and 4 (6.7%) had grade 2 dysfunction. Systolic dysfunction (LVEF <50%) was present in 10 patients (16.7%), confirming that diastolic dysfunction was more than three times as prevalent as systolic dysfunction in this cohort (Table 3).

 The mean GSPECT-derived parameters were: PFR 2.07±0.78 EDV/s, TTPFR 242.32±31.47 ms, and LVEF 55±7.05%.

**Table 3:** Grouping of patients based on echocardiography results

**Variable**	**Category**	**F (%)**
Diastolic function	Normal	29 (48.3%)
	Grade 1	27(45%)
	Grade 2	4 (6.7%)
Systolic function	Normal	50 (83.3%)
	Systolic Dysfunction	10 (16.7%)

### Correlation between GSPECT and Echocardio-graphy

 A significant correlation was observed between the ischemia pattern on GSPECT and the severity of diastolic dysfunction on echocardiography (p=0.01, Table 4). Patients with more severe diastolic dysfunction tended to have more pronounced perfusion defects.

 Analysis of GSPECT diastolic parameters against risk factors revealed that a significantly lower PFR was associated with male gender (1.8 vs. 2.2 EDV/s, p=0.002), age >60 years (2.0 vs. 2.3 EDV/s, p=0.015), and smoking (1.9 vs. 2.2 EDV/s, p=0.047). No significant differences were found for TTPFR across these groups. (Table 5).

**Table 4:** Distribution of the perfusion index obtained from the scan in diastolic function groups

**Diastolic function**	**Heart perfusion index of scan**			
	**Normal**	**Mild ischemia**	**Moderate ischemia**	**Severe ischemia**
Normal	22	6	0	1
Grade 1	4	9	12	2
Grade 2	0	0	2	2

**Table 5:** Comparison of PFR and TTPFR in groups with and without heart risk factors

**Risk factor**	**Category**	**PFR**		**TPFR**	
		**Mean±SD**	***P*** ** value**	**Mean±SD**	***P*** ** value**
Age	<60	2.3±1.0	0.015	238.3±51.6	0.1
	>60	2.0±1.0		244.9±162.5	
BMI	<30	2.07±0.9	0.933	238.6±47.6	0.394
	>30	2.07±1.1		249.3±62.9	
Gender	Man	1.8±0.9	0.002	230.5±62.3	0.13
	Female	2.2±1.1		251.3±50.5	
HTN	Yes	2.07±1.1	0.779	244.9±61.3	0.302
	No	2.07±0.9		232.3±44.4	
Smoking	Yes	1.9±0.7	0.047	233.1±36.2	0.693
	No	2.2±1.0		248.6±57.5	
Diabetes control	Yes	2.1±1.4	0.496	248.3±50.5	0.13
	No	1.9±0.8		230.5±62.3	

### Diagnostic Accuracy of GSPECT Parameters

 The diagnostic performance of GSPECT parameters for identifying LVDD (any grade) is summarized in Table 6.

• PFR (<2.5 EDV/s) showed the best balance, with a sensitivity of 87%, specificity of 48.2%, and overall accuracy of 68.3%.

• TTPFR (>180 ms) demonstrated high sensitivity (90.3%) but very low specificity 

(17.2%), resulting in an accuracy of 55%.

• LVEF was highly specific (96.5%) but insensitive (19.3%) for detecting diastolic dysfunction.

**Table 6:** Diagnostic value of PFR, TTPFR, and EF compared to the diastolic index obtained from echocardiography

**Index**	**N**	**Index**	**Diagnostic value** ** (%)**
True positive	27	Sensitivity	87%
True negative	14	Specificity	48.2%
False positive	15	Positive news value	64.2%
False negative	4	Negative news value	77.7%
TTPFR	N	Index	Diagnostic value (%)
True positive	28	Sensitivity	90.3%
True negative	5	Specificity	17.2%
False positive	24	Positive news value	53.8 %
False negative	3	Negative news value	62.5%
EF (scan)	N	Index	Diagnostic value (%)
True positive	6	Sensitivity	19.3%
True negative	28	Specificity	96.5%
False positive	1	Positive news value	85.7%
False negative	25	Negative news value	52.8%

When considering the GSPECT study as a whole (integrating perfusion and functional data), the overall accuracy for diagnosing LVDD was 63.3%, with a sensitivity of 77.4% and specificity of 48.2% (Table 7).

**Table 7:** The diagnostic value of Gated SPECT scanning compared to echocardiography in diagnosing left ventricular diastolic dysfunction

**Index**	**N**	**Index**	**Diagnostic value (%)**
True positive	26	Sensitivity	77.4%
True negative	15	Specificity	48.2%
False positive	14	Positive news value	61.5%
False negative	5	Negative news value	66.6%

## Discussion

 This study demonstrates that diastolic parameters derived from GSPECT, particularly the PFR, have significant value in identifying LVDD in diabetic patients without known cardiac disease. Our findings confirm a high prevalence of subclinical diastolic dysfunction in this population, detectable by both echocardiography and GSPECT.

 The key finding is the high sensitivity (87%) of a reduced PFR for detecting echocardio-graphically proven LVDD. This suggests that a normal PFR on GSPECT could be a useful tool to rule out significant diastolic dysfunction in this patient group. The specificity of PFR was moderate (48.2%), indicating that other factors might influence this parameter. The high sensitivity but low specificity of TTPFR limits its standalone diagnostic utility. The strong association between lower PFR and traditional risk factors like older age, male gender, and smoking further supports its role as a marker of cumulative cardiovascular risk.

 Our results are partially consistent with previous work. Malek et al. (10) also reported a significant correlation between GSPECT diastolic indices and echocardiography, though with different sensitivity and specificity values (70% and 60% for PFR, respectively). This discrepancy may be attributable to differences in sample size, patient population, or the echocardiographic criteria used for defining LVDD. Our study utilized more contemporary guidelines for echocardiographic classification, which may provide a more robust reference standard.

 Similar to the conclusions of Bennett et al. (11), we found a significant correlation between GSPECT-derived parameters and diastolic function. Our data support their recommendation to incorporate diastolic analysis into routine GSPECT reporting. In contrast, Dabbagh et al. (12) found no significant correlation, a divergence that may be explained by methodological differences, including their smaller sample size and the specific echocardiographic parameters chosen for comparison.

 The significant correlation between perfusion defects and diastolic dysfunction grade underscores the interplay between ischemia 

and myocardial relaxation abnormalities. This highlights the unique advantage of GSPECT: the ability to simultaneously evaluate for coronary artery disease and its functional consequences on diastolic performance in a single study.

### Limitations

 This study has several limitations. The single-center design and relatively small sample size may limit the generalizability of the findings. The convenience sampling method could introduce selection bias. The cross-sectional nature of the study precludes assessment of the prognostic value of these GSPECT parameters. Future multi-center, longitudinal studies with larger cohorts are needed to confirm these findings and establish the prognostic implications of abnormal diastolic indices on GSPECT.

## Conclusion

 In diabetic patients undergoing myocardial perfusion imaging, Gated SPECT provides valuable incremental information for the detection of subclinical left ventricular diastolic dysfunction. The peak filling rate (PFR) demonstrates high sensitivity, making it a useful screening parameter. We recommend the routine calculation and reporting of diastolic function indices, particularly PFR, in GSPECT studies of diabetic patients. This practice could enhance the early diagnosis of diabetic cardiomyopathy, allowing for prompt intervention and improved patient management.
